# Prim-O-glucosylcimifugin enhances the antitumour effect of PD-1 inhibition by targeting myeloid-derived suppressor cells

**DOI:** 10.1186/s40425-019-0676-z

**Published:** 2019-08-28

**Authors:** Wanfeng Gao, Xiaoyun Zhang, Wendong Yang, Daolei Dou, Heng Zhang, Yuanhao Tang, Weilong Zhong, Jing Meng, Yun Bai, Yanrong Liu, Lan Yang, Shuang Chen, Huijuan Liu, Cheng Yang, Tao Sun

**Affiliations:** 1 State Key Laboratory of Medicinal Chemical Biology and College of Pharmacy, Nankai University, Haihe Education Park, 38 Tongyan Road, Tianjin, 300353 China; 2 College of Life Sciences, Nankai University, Tianjin, China; 3 Tianjin Key Laboratory of Early Druggability Evaluation of Innovative Drugs and Tianjin Key Laboratory of Molecular Drug Research, Tianjin International Joint Academy of Biomedicine, Tianjin, China; 4 Department of Experimental Facility, State Key Laboratory of Medical Chemical Biology, Nankai University, Tianjin, China; 5 Academy of Mathematics and Systems Science, Chinese Academy of Sciences School of Economics and Management, University of Chinese Academy of Sciences, Beijing, China

**Keywords:** Prim-O-glucosylcimifugin, Myeloid-derived suppressor cells, Proliferation, Metabolism, PD-1 inhibitor

## Abstract

**Background:**

Myeloid-derived suppressor cells (MDSCs) are immunosuppressive cells that play an important role in immune evasion, PD-1/PD-L1 inhibitor tolerance and tumour progression. Therefore, MDSCs are potential targets for cancer immunotherapy. In this study, we screened an effective polymorphonuclear MDSC (PMN-MDSC) inhibitor from the Traditional Chinese Medicine Library and evaluated its synergistic antitumour effects with PD-1 inhibitor.

**Methods:**

In the present study, we found that PMN-MDSCs accumulate heavily in the spleen and bone marrow of melanoma (B16-F10) tumour-bearing mice. Then, we determined the top 10 key proteins in the upregulated KEGG pathways of PMN-MDSCs in tumour-bearing mice through proteomics and Cytoscape analysis. The key proteins were then used as targets for the screening of PMN-MDSC inhibitors from the traditional Chinese Medicine Library (20000 compounds) through molecular docking and weight calculation of the docking score. Finally, the inhibitory effect of the inhibitor was verified through proteomics and metabolomics analysis in vitro and melanoma (B16-F10) and triple-negative breast cancer (4 T1) mouse tumour models in vivo.

**Results:**

Traditional Chinese medicine saposhnikovia root extract Prim-O-glucosylcimifugin (POG) could bind well to the target proteins and inhibit the proliferation, metabolism and immunosuppressive ability of PMN-MDSCs by inhibiting arginine metabolism and the tricarboxylic acid cycle (TCA cycle). POG could also increase CD8 T-lymphocyte infiltration in the tumours and enhance the antitumour effect of PD-1 inhibitor in B16-F10 and 4 T1 mouse tumour models.

**Conclusions:**

POG was successfully screened from the traditional Chinese Medicine library as a PMN-MDSC inhibitor. POG exhibited a good synergistic antitumour effect with PD-1 inhibitor. This study provided a potential option for enhancing the efficacy of PD-1 inhibitors in clinical applications.

**Electronic supplementary material:**

The online version of this article (10.1186/s40425-019-0676-z) contains supplementary material, which is available to authorized users.

## Introduction

Recent human clinical trials have shown that PD-1/PDL-1 inhibitors exert good antitumour effects, and PD-1/PDL-1 inhibitors were approved by the FDA for multiple tumour types. However, most cancer patients exhibit poor response to PD-1/PDL-1 inhibitors [1]. In triple-negative breast cancer cells (4 T1), the overall response rates of PD-1/PDL-1 inhibitors are only 5–30%; in melanoma (B16-F10), tumour recurrence often occurs despite continuous treatment after the initial tumour subsides [2, 3]. The limitation of immune checkpoint inhibitors may be mediated by the immunosuppressive tumour microenvironment, which mainly includes some immunosuppressive factors released by tumours and some infiltrating immunosuppressive cells, such as regulatory T-lymphocytes (Tregs) and myeloid-derived suppressor cells (MDSCs) [4–6].

MDSCs are abundant in the lymphoid organs of tumour-bearing mice and patients. MDSCs mainly include polymorphonuclear MDSC (PMN-MDSC) and monocytic MDSC (M-MDSC) subpopulations. M-MDSCs are labelled as CD11b^+^Ly6G^−^Ly6C^high,^ and PMN-MDSCs are labelled as CD11b^+^Ly6G^+^Ly6C^low^ in mice. In humans, M-MDSCs are labelled as HLA-DR^−^CD11b^+^CD33^+^CD14^+^, and PMN-MDSCs are labelled as HLA-DR^−^CD11b^+^CD33^+^CD15^+^ [7]. MDSCs respond to the stimulation of cancer-derived factors, such as granulocyte colony-stimulating factor (G-CSF), interleukin-6 (IL-6) and granulocyte monocyte colony-stimulating factor (GM-CSF), through transcription factors STAT1, STAT3, STAT6 and NF-κB to proliferate and obtain immunosuppressive activity in bone marrow [8, 9]. Activated MDSCs are recruited to tumour sites through the actions of inflammatory factors (i.e., IL6 and IL1β), PEG2, S1P and chemokines (i.e., CCL2) [10–12]. In the tumour sites, MDSCs form the immunosuppressive microenvironment by producing Arg-1, iNOS, IDO, NOX2 and immunosuppressive cytokines [13, 14], and MDSCs express a large amount of PD-L1 through the stimulation of hypoxia-inducible factor 1α (HIF1α) and tumour-derived exosomes and eventually inhibit the activity of T cells [15, 16]. In addition, activated MDSCs in tumours could affect tumour remodelling and tumour angiogenesis by producing VEGF, basic fibroblast growth factor (bFGF), Bv8 and MMP9, thereby promoting tumour progression [17, 18]. Therefore, targeting MDSCs is a new cancer treatment strategy that could enhance the antitumour effects of PD-1/PD-L1 inhibitors.

In the present study, we successfully screened prim-O-glucosylcimifugin (POG) as a PMN-MDSC inhibitor from the traditional Chinese Medicine library. In vitro and in vivo experiments showed that POG could inhibit the proliferation, metabolism and immunosuppressive ability of PMN-MDSCs, improve the tumour immunosuppressive microenvironment and generate a synergistic effect with PD-1 inhibitors in B16-F10 and 4 T1 mouse tumour models. This finding suggested that POG is a sensitiser for PD-1 inhibitors.

## Materials and methods

### Tissue processing and flow cytometry

Bone marrow cells were flushed from the femurs and tibias with PBS with a syringe. The spleen samples were processed through mechanical dissociation, and tumour tissues were processed into single-cell suspensions by dissociating the tissues enzymatically for 1 h with 1 mg/ml type I collagenase (Sigma-Aldrich) in the presence of 50 units/mL DNase (Sigma-Aldrich). The cells were lysed with red blood cell lysis buffer and filtered with a 100 μm membrane, further washed with 1% BSA in PBS and blocked by non-specific staining with Fc Block (anti-mouse CD16/32 mAb; BD Biosciences). The samples were then stained with fluorescence-conjugated antibodies against the surface markers CD45 (clone 30-F11, eBioscience), CD11b (clone M1/70, eBioscience), Ly6C (clone HK1.4, eBioscience), Ly6G (clone 1A8-Ly6g, eBioscience), CD3 (clone 145-2C11, eBioscience) and CD8 (clone 53–6.7, eBioscience) and detected using flow cytometry (LSR BD Fortessa).

### Cell sorting of PMN-MDSCs and T-lymphocytes

The single-cell suspensions of the tumour, bone marrow and spleen samples were stained with fluorescence-conjugated antibodies against the surface markers CD11b, Ly6C, Ly6G, CD3 and CD8 for 30 min at 4 °C. PMN-MDSCs and T-lymphocytes were then sorted through flow cytometry (BD AriaIII). The sorted PMN-MDSCs were cultured in RPMI 1640 with 10% foetal bovine serum, 20 ng/mL recombinant GM-CSF (Recombinant CJ46, Novoprotein), 20 ng/mL IL6 (CG39, Novoprotein) and 50 μM 2-mercaptoethanol (60–24-2, Biotech). The sorted T-lymphocytes were cultured in RPMI 1640 with 10% foetal bovine serum and stimulated with CD3 (clone 145-2C11, eBioscience) and CD28 antibodies (clone 37.51, eBioscience, 5 μg/mL).

### PMN-MDSC isolation and proteomic analysis

Bone marrow cells were harvested from naive C57BL6 mice and B16-F10 tumour-bearing mice and then processed into single-cell suspensions. Naive PMN-MDSCs and B16-F10 tumour-bearing PMN-MDSCs were sorted through flow cytometry. The sorted naive PMN-MDSCs and B16-F10 tumour-bearing PMN-MDSCs were then prepared for proteomics analysis. A fold change of more than 2 was defined as significantly different. Gene ontology (GO) analysis and KEGG enrichment analysis were performed using the DAVID database [19, 20]. Protein–protein interaction networks were analysed with the STRING database [21].

### Screening PMN-MDSC inhibitors by molecular docking and weight calculation of docking scores

To screen the natural inhibitors of PMN-MDSCs, we performed Cytoscape analysis on the proteins in the upregulated KEGG pathways of the PMN-MDSCs in B16-F10 tumour-bearing mice, and the top 10 key proteins were obtained according to the degree in the protein–protein interaction networks analysed with Cytoscape. The natural inhibitors of PMN-MDSCs were then screened from the traditional Chinese Medicine library (20000 compounds) by targeting the top 10 key proteins with molecular docking. The structures of the traditional Chinese medicines were downloaded from TCM Database@Taiwan (http://tcm.cmu.edu.tw/) [22, 23] and showed in Additional file 1. The compounds with docking score absolute values of more than 4 for all the targets were selected as candidate compounds. Finally, the weight calculation of the candidate compounds was performed according to the degree of the top 10 key proteins in Cytoscape analysis and the compound docking scores with the top 10 key proteins by using the formula proteins degree × the compound docking scores. The effects of the first five compounds on PMN-MDSCs were verified in vitro. All results are expressed as the mean ± SD.

### In vivo experiment

B16-F10 cells and 4 T1 cells were purchased from KeyGen Biotech (Nanjing, China). The cells were cultured with RPMI 1640 (HyClone) with 10% foetal bovine serum (HyClone). Female C57BL/6 and BALB/C mice (6 weeks old) were purchased from the Animal Centre of the Academy of Military Medical Sciences (Beijing, China) and maintained in a temperature-controlled room with a 12 h/12 h light/dark schedule. All animal experiments conformed to the guidelines of the Animal Ethics Committee of the Tianjin International Joint Academy of Biotechnology and Medicine. To establish the B16-F10 tumour model, we resuspended 4 × 10^5^ cells in 0.1 mL PBS, and the suspensions were subcutaneously injected into the right lateral flank of the C57BL/6 mice. After the tumour sizes reached 120–180 mm^3^, the animals were randomly assigned to six groups (*n* = 6): control, POG, 1H-indole-3-carboxylic acid, tetrahydrofolate, okanin and 6-methoxy-2-benzoxazolinone groups. The control group only received the vehicle (5% DMSO in 20% hydroxypropyl beta-cyclodextrin buffer). The POG (Push bio-technology, PS00838), 1H-indole-3-carboxylic acid (SIGMA-ALDRICH, 284734), tetrahydrofolate (SIGMA-ALDRICH, T3125), okanin (YUANYE, JO515750) and 6-methoxy-2-benzoxazolinone (SIGMA-ALDRICH, 543551) treatments were performed by intraperitoneal injection (100 mg/kg/day) for 14 days. To evaluate the dose dependence of POG, we randomly assigned the animals when the tumour sizes reached 120–180 mm^3^ to three groups (*n* = 6), namely, control, POG-low and POG-high groups, which were administered vehicle (5% DMSO in 20% hydroxypropyl beta-cyclodextrin buffer) or 100 or 200 mg/kg/day POG intraperitoneally for 14 days. Tumour volume was measured every 3 days. Tumour volume was calculated as length × width^2^ / 2.

### Cell apoptosis assay

To determine the cytotoxic effect of POG on PMN-MDSCs, CD8 T-lymphocytes and B16-F10 cells, we sorted PMN-MDSCs and CD8 T-lymphocytes from the bone marrow and spleen of B16-F10 tumour-bearing mice, and the cells were cultured in MDSCs and T-lymphocyte media, respectively. The PMN-MDSCs, CD8 T-lymphocytes and B16-F10 cells were then divided into three groups: control, POG (50 μM) and POG (100 μM) groups. After 48 h, the cells were stained with an Annexin V/PI apoptosis detection kit (KeyGen Biotech, China) and analysed by flow cytometry after the cells were incubated in the dark for 30 min. All results are expressed as the mean ± SD.

### Cell proliferation assay

To determine the effect of POG on PMN-MDSCs, CD8 T-lymphocytes and B16-F10 cells, we sorted PMN-MDSCs and CD8 T-lymphocytes from the bone marrow and spleen of the B16-F10 tumour-bearing mice, and the cells were cultured in MDSCs and T-lymphocyte media, respectively. PMN-MDSCs, CD8 T-lymphocytes and B16-F10 cells were stained with carboxyfluorescein succinimidyl ester (CFSE; Sigma), and PMN-MDSCs, CD8 T-lymphocytes and B16-F10 cells were divided into three groups, namely, control, POG (50 μM) and POG (100 μM) groups. After 48 h POG treatment, the CFSE dilution was determined using flow cytometry analysis [24, 25]. All the results are expressed as the mean ± SD.

### Proteomic and metabolomic analysis

To determine the effect of POG on PMN-MDSCs, we sorted PMN-MDSCs from the bone marrow of B16-F10 tumour-bearing mice, and the cells were cultured in MDSC medium. The PMN-MDSCs were then divided into two groups, namely, the control and POG (100 μM) groups. After 48 h, the cells were detected by proteomics analysis and UHPLC-QE-MS non-target metabolomics analysis. A fold change of more than 2 or 1.5 is defined as significantly different.

### Quantitative real-time PCR

PMN-MDSCs were sorted from the bone marrow of the B16-F10 tumour-bearing mice, cultured in MDSC medium, and then divided into three groups, namely, the control, POG (50 μM) and POG (100 μM) groups. After 48 h, the cells were harvested to examine the effect of POG on the expression of iNOS and Arg-1 in the PMN-MDSCs by using qRT-PCR. Total RNA was extracted from the PMN-MDSCs by using TRIzol reagent (Invitrogen, USA) in accordance with the manufacturer’s instructions. cDNA was synthesised from the total RNA by using a PrimeScript RT reagent kit (Tiangen, China). U6 was used as an internal control. The primers used for the target genes were GAPDH 5′-AACTTTGGCATTGTGGAAGG-3′ and 5′- ACACATTGGGGGTAGGAACA-3′; iNOS, 5′-AACGGAGAACGTTGGATTTG-3′ and 5′-CAGCACAAGGGGTTTTCTTC-3′; and Arg1, 5′-GCTGTCTTCCCAAGAGTTGGG-3′ and 5′- ATGGAAGAGACCTTCAGCTAC-3′. All results are expressed as the mean ± SD.

### Western blot analysis

PMN-MDSCs were sorted from the bone marrow of the B16-F10 tumour-bearing mice, cultured in MDSC culture medium, and divided into three groups, namely, control, POG (50 μM) and POG (100 μM) groups. After 48 h, the cells were harvested, and the effect of POG on the expression of iNOS and Arg-1 in the PMN-MDSCs was determined by Western blot analysis. The cells were then washed with PBS and lysed in ice-cold lysis buffer with protease inhibitor cocktail (Sigma) for 30 min. The lysates were separated through SDS-PAGE and then transferred to PVDF membranes (Millipore, Bedford, MA, USA). The membranes were blocked and incubated with primary antibody Arg-1 (Affinity Bioreagents, USA) and iNOS (Affinity Bioreagents, USA). The membranes were incubated with the second antibody (Santa Cruz Biotechnology, USA). GAPDH was used as the loading control. Protein expression was detected with an enhanced chemiluminescence detection kit (Millipore, USA). Densitometric analysis was performed with ImageJ software. All results are expressed as the mean ± SD.

### ARG-1, ROS and NO measurements

PMN-MDSCs were sorted from the bone marrow of the B16-F10 tumour-bearing mice, cultured in MDSC culture medium, and divided into three groups, namely, control, POG (50 μM) and POG (100 μM) groups. After 48 h, the cells were harvested. ARG1 activity, ROS and NO were detected by using an ARG1 activity assay kit (Abcam), DCFDA (Invitrogen) and a Griess reagent system (Promega) in accordance with the manufacturer’s instructions. All results are expressed as the mean ± SD.

### T-lymphocyte proliferation assay

T-lymphocytes sorted from the spleens of the B16-F10 tumour-bearing mice were cultured in T-lymphocyte medium and stained with CFSE (Sigma). After the cells were co-cultured with PMN-MDSCs or M-MDSCs for 48 h, the cells were stained for surface markers with CD8 antibody (clone 53–6.7, eBioscience). The CFSE dilution in CD8 T-lymphocytes was determined through flow cytometry analysis [24, 25]. All results are expressed as the mean ± SD.

### IFN-γ production assays

T-lymphocytes sorted from mouse spleens were cultured in T-lymphocyte medium with or without POG. After 48 h, supernatant IFN-γ levels were quantified by ELISA (eBioscience) in accordance with the manufacturer’s instructions. All the results are expressed as the mean ± SD.

### Effect of POG combined with PD-1 inhibitor in vivo

The B16-F10 tumour model was established by using the method described above. To establish the 4 T1 tumour model, we injected the resuspended 4 × 10^5^ 4 T1 cells in 0.1 mL PBS into the fourth pair of the mammary fat pad of BALB/C mice. When the tumour volumes of B16-F10 and 4 T1 tumour-bearing mice reached 120–180 mm^3^, the mice were randomly distributed into the following groups (*n* = 6): control, POG, anti-PD-1 (Bio X Cell RPM1–14, rat IgG2a) and a combination of POG and anti-PD-1 groups. The control group was treated with the vehicle alone (5% DMSO in 20% hydroxypropyl beta-cyclodextrin buffer). The POG group was administered intraperitoneally daily at 100 and 200 mg/kg for 14 days. Anti-PD-1 antibody (clone RMP1–14, Bio X Cell) or isotype control antibody (clone 2A3, Rat IgG2a, Bio X Cell) was intraperitoneally given on days 11, 14, 17, 20 and 23 (200 μg/injection). Tumour volume was measured every 3 days. Tumour volume was calculated as length × width^2^ / 2.

### Statistical analysis

All statistical analyses were performed with GraphPad Prism7 software for Windows. Statistically significant differences were calculated by using Student’s t-test. Overall survival analysis was performed by using the Kaplan–Meier method with the log-rank test, and a *p* value of < 0.05 was considered statistically significant.

## Results

### More PMN-MDSCs accumulated in B16-F10 tumour-bearing mice than in naive mice

**Fig. 1:**
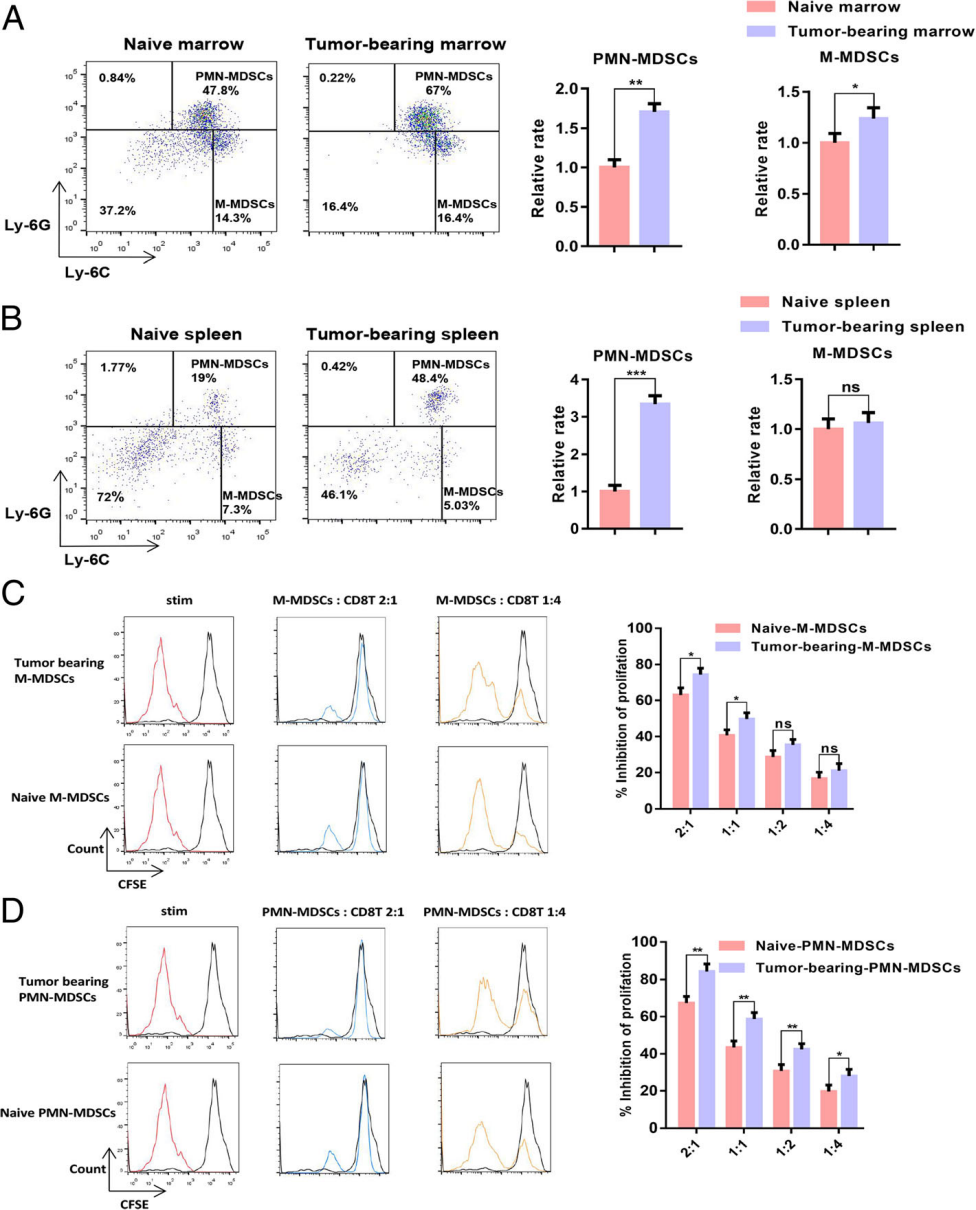
PMN-MDSCs accumulated in B16-F10 tumour-bearing mice in contrast to those in naive mice. **a** Dotplots of live CD11b^+^ cells in the bone marrow of naive or B16-F10 tumour-bearing mice (left panels) and relative proportions of PMN-MDSCs (CD11b^+^Ly6G^+^Ly6C^low^) and M-MDSCs (CD11b^+^Ly6G^−^Ly6C^high^) in the bone marrow of naive and B16-F10 tumour-bearing mice (right charts). **b** Dotplots of live CD11b^+^ cells in the spleens of naive mice or B16-F10 tumour-bearing mice (left panels), and relative proportions of PMN-MDSCs (CD11b^+^Ly6G^+^Ly6C^low^) and M-MDSCs (CD11b^+^Ly6G^−^Ly6C^high^) in the spleens of naive and B16-F10 tumour-bearing mice (right charts). **c**–**d** Dose-dependent suppression of CD8 T-lymphocyte proliferation by sorted bone marrow M-MDSCs and PMN-MDSCs. Representative CFSE histograms are shown (unstimulated CFSE-labelled T-lymphocytes in black). The pooled data from three independent experiments are shown. All data are represented as the mean ± SD. * *p* < 0.05, ** *p* < 0.01, ***p < 0.001, ****p < 0.0001

When the tumour volume reached 1000 mm^3^, the naive mice and B16-F10 tumour-bearing mice were sacrificed, and the proportion of MDSCs in the spleen and bone marrow samples was measured. The results showed that the proportion of MDSCs in the spleen and bone marrow samples of the B16-F10 tumour-bearing mice considerably increased relative to the proportion in the naive mice. The CD11b^+^Ly-6G^+^ Ly-6C^low^ PMN-MDSC population in the bone marrow and spleen samples of the B16-F10 tumour-bearing mice increased more significantly than the CD11b^+^Ly-6G^−^ Ly-6C^high^ M-MDSC population (Fig. 1a–b). We sorted naive PMN-MDSCs, B16-F10 tumour-bearing PMN-MDSCs, naive M-MDSCs and B16-F10 tumour-bearing PMN-MDSCs and then co-cultured these cells with CD8 T-lymphocytes at 4:1, 2:1, 1:1 and 1:2. The results of T-lymphocyte proliferation experiments showed that the ability of PMN-MDSCs to inhibit CD8 T-lymphocyte proliferation is stronger than that of M-MDSCs in B16-F10 tumour-bearing mice (Fig. 1c–d).

### Differentially expressed genes of PMN-MDSCs in tumour-bearing mice are mainly enriched in proliferation and metabolism-related pathways

**Fig. 2:**
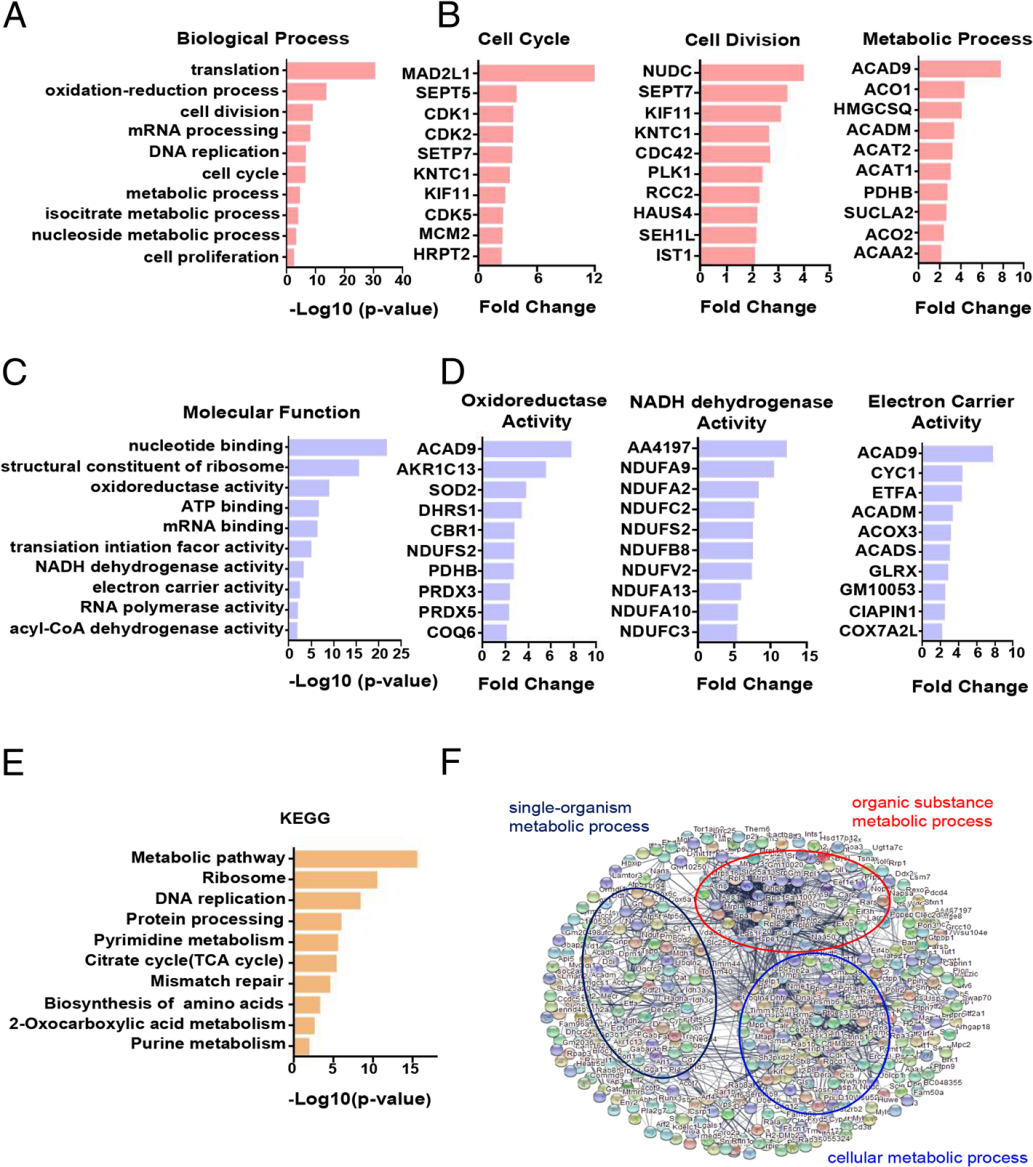
Differentially expressed genes of PMN-MDSCs in tumour-bearing mice are mainly enriched in proliferation and metabolism-related pathways. **a** Statistical analysis of upregulated biological processes of B16-F10 tumour-bearing PMN-MDSCs. **b** The upregulated genes associated with the cell cycle, cell division and metabolic process of B16-F10 tumour-bearing PMN-MDSCs. **c** Statistical analysis of the upregulated molecular function of B16-F10 tumour-bearing PMN-MDSCs. **d** The upregulated genes associated with oxidoreductase, NADH dehydrogenase and electron carrier activities. **e** KEGG analysis of the upregulated genes of B16-F10 tumour-bearing PMN-MDSCs (**f**). The protein–protein interaction networks of upregulated proteins of B16-F10 tumour-bearing PMN-MDSCs. Significantly changed proteins are correlated with cell metabolism

The PMN-MDSCs sorted from the bone marrow of the naive and B16-F10 tumour-bearing mice were collected for proteomic analysis and analysed by the DAVID database. The results of GO analysis showed that the upregulated genes of PMN-MDSCs in tumour-bearing mice were enriched in the function of proliferation and metabolism compared with PMN-MDSCs in naive mice. The enhanced functions included cell cycle, cell division, metabolic process-related biological processes (Fig. 2a) and oxidoreductase activity, NADH dehydrogenase activity and electron carrier activity-related molecule function (Fig. 2c). The upregulated genes associated with the cell cycle, cell division and metabolic process in the B16-F10 tumour-bearing PMN-MDSCs are shown in Fig. 2b. The upregulated genes associated with oxidoreductase, NADH dehydrogenase and electron carrier activities in the B16-F10 tumour-bearing PMN-MDSCs are shown in Fig. 2d. The KEGG analysis showed that the upregulated genes of PMN-MDSCs in B16-F10 tumour-bearing mice were enriched in cell proliferation and metabolic pathways, such as the metabolic pathways, tricarboxylic acid cycle (TCA cycle) and DNA replication (Fig. 2e). Furthermore, we analysed the protein-protein interaction of the upregulated differential genes of B16-F10 tumour-bearing PMN-MDSCs by using the STRING database. The results showed that the upregulated genes were mainly related to cell metabolism (Fig. 2f).

### POG, a natural inhibitor of PMN-MDSCs, was screened using molecular docking and weight calculation of docking scores

**Fig. 3:**
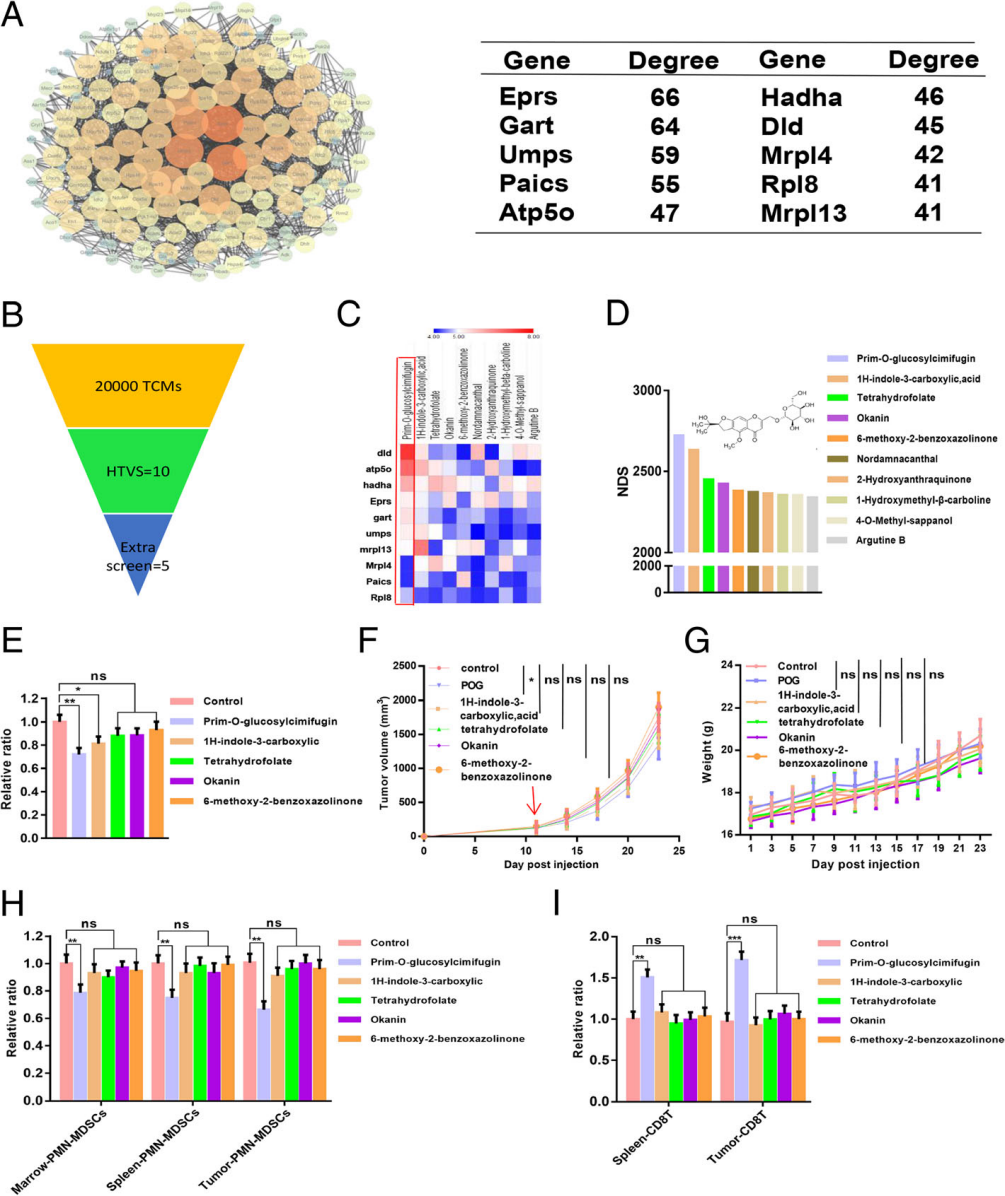
POG, as a natural inhibitor of PMN-MDSCs, is screened by molecular docking and weight calculation of docking scores. **a** Cytoscape analysis of the proteins in the upregulated KEGG pathway of B16-F10 tumour-bearing PMN-MDSCs and the top 10 key proteins of upregulation proteins of B16-F10 tumour-bearing PMN-MDSCs obtained according to the Cytoscape analysis degree. **b** Screening result of PMN-MDSC inhibitors from the traditional Chinese Medicine library with the top 10 key proteins as targets by molecular docking and weight calculation of docking score. **c** The 10 compounds from the traditional Chinese Medicine library, which binds well with 10 key proteins, and the absolute value of docking scores are more than 4 with all 10 proteins. **d** Weight calculation of the 10 compounds from the traditional Chinese Medicine library. **e** Inhibitory effect of the top 5 compounds on PMN-MDSCs (CD11b^+^ Ly6G^+^ Ly6C^low^) in vitro. **f** The tumour growth curves of B16-F10 tumour-bearing mice after the top 5 compound treatments (*n* = 6). **g** Body weight of B16-F10 tumour-bearing mice after the top 5 compound treatments (n = 6). **h** Relative proportion of PMN-MDSCs (CD11b^+^ Ly6G^+^ Ly6C^low^) in bone marrow, spleen and CD45^+^ cells from tumours of control and top 5 compound-treated B16-F10 tumour-bearing mice (*n* = 6). **i** Relative proportion of CD8 T-lymphocytes (CD3^+^CD8^+^) in spleens and CD45^+^ cells from tumours of control and top 5 compound-treated B16-F10 tumour-bearing mice (*n* = 6). The pooled data from three independent experiments are shown. All data are represented as the mean ± SD. * *p* < 0.05, ** *p* < 0.01, ***p < 0.001, ****p < 0.0001

Based on the results of proteomic analysis, we found that the major enhancement pathways of the PMN-MDSCs in B16-F10 tumour-bearing mice were related to proliferation and metabolism. We then screen the key proteins in these pathways and the inhibitors that repressed these pathways by targeting the key proteins. We performed Cytoscape analysis of the proteins in the upregulated KEGG pathways and then ranked the top key 10 proteins in these pathways in accordance with the degree level in the Cytoscape analysis (Fig. 3a). We then screened natural inhibitors of MDSCs from the traditional Chinese Medicine library by targeting the top 10 key proteins with molecular docking. The compounds with a docking score absolute value with all 10 key proteins of more than 4 were selected as candidate inhibitors (Fig. 3b–c). The structure of 10 candidate inhibitors could be found in the Appendix. We then performed weight calculations of candidate inhibitors to sort the candidate inhibitors (Fig. 3d). Furthermore, we verified the inhibitory activities of the top 5 candidate inhibitors, namely, POG, 1H-indole-3-carboxylic acid [26], tetrahydrofolate [27], okanin [28] and 6-methoxy-2-benzoxazolinone [29], on PMN-MDSCs in vitro and in vivo. In vitro, bone marrow cells from the B16-F10 tumour-bearing mice were treated with the vehicle control and 100 μM of the top 5 compounds. After 48 h, we evaluated the percentages of PMN-MDSCs in bone marrow cells by flow cytometry and found that POG exhibited the best inhibitory effect on PMN-MDSCs (Fig. 3e). In vivo, a B16-F10 subcutaneous tumour model in C57BL6 mice was established for the evaluation of the antitumour effects of the top five compounds. We found that POG exhibited the best antitumour effect at a dose of 100 mg/kg and reduced the proportion of PMN-MDSCs in the bone marrow, spleen and CD45^+^ cells in tumours (Fig. 3f–h). POG also increased the number of CD8 T-lymphocytes in the spleens and CD45^+^ cells in tumour samples at a dose of 100 mg/kg. (Fig. 3i).

### POG inhibits the proliferation and metabolism of PMN-MDSCs in vitro

**Fig. 4:**
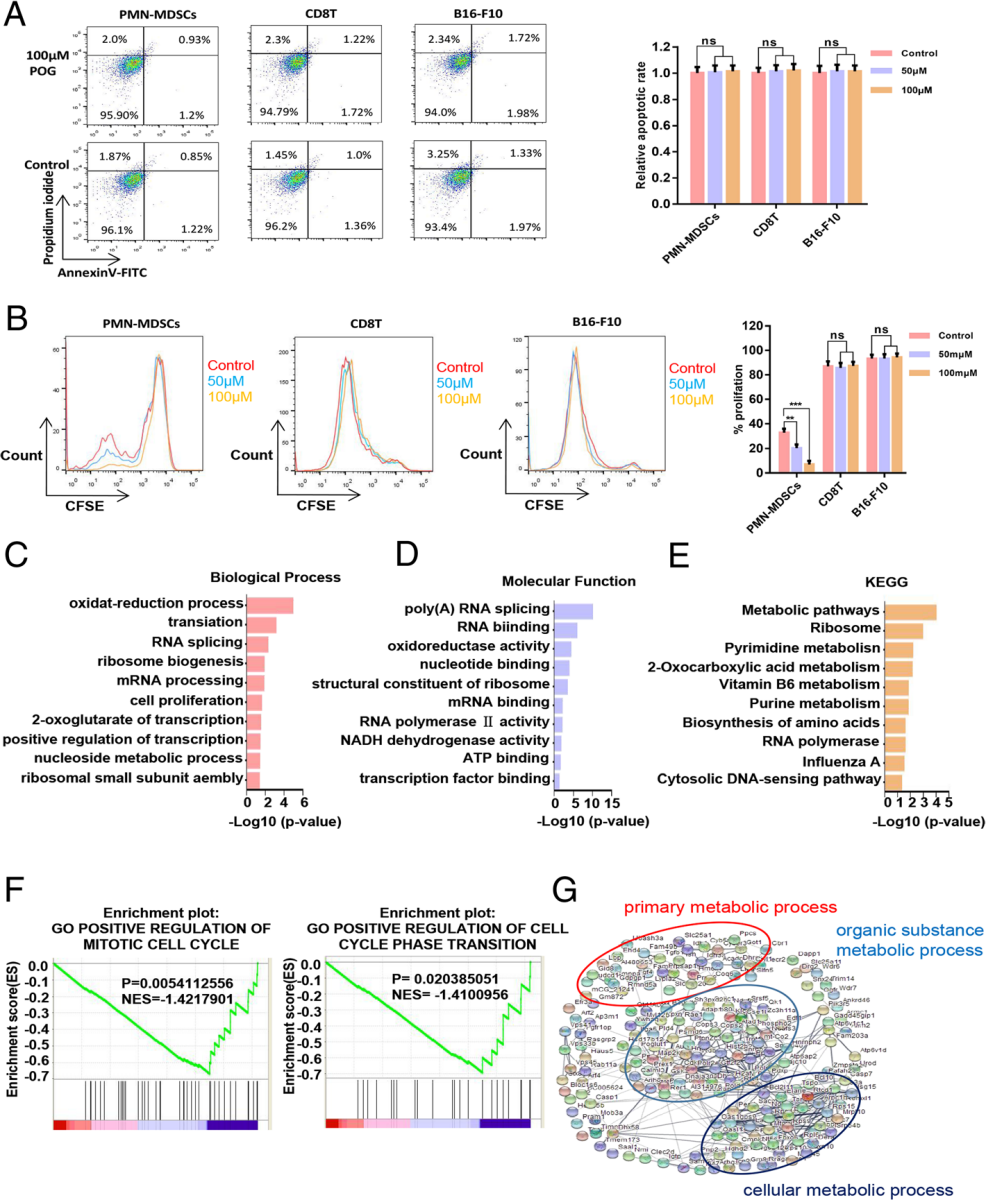
POG inhibits the proliferation and metabolism of PMN-MDSCs in vitro. **a** The cytotoxic effect of POG on PMN-MDSCs, CD8 T-lymphocytes and B16-F10 cells. **b** Effect of POG on the proliferation of PMN-MDSCs, CD8 T-lymphocytes and B16-F10 cells. **c** Statistical analysis of downregulated biological processes of B16-F10 tumour-bearing PMN-MDSCs after POG treatment. **d** Statistical analysis of the downregulated molecular function of B16-F10 tumour-bearing PMN-MDSCs after POG treatment. **e** KEGG analysis of the downregulated genes of B16-F10 tumour-bearing PMN-MDSCs after POG treatment. **f** GSEA analysis of the downregulated genes of B16-F10 tumour-bearing PMN-MDSCs after POG treatment. **g** The protein–protein interaction networks of downregulated proteins of B16-F10 tumour-bearing PMN-MDSCs after POG treatment. The pooled data from three independent experiments are shown. All data are represented as the mean ± SD. * *p* < 0.05, ** *p* < 0.01, ***p < 0.001, ****p < 0.0001

**Fig. 5:**
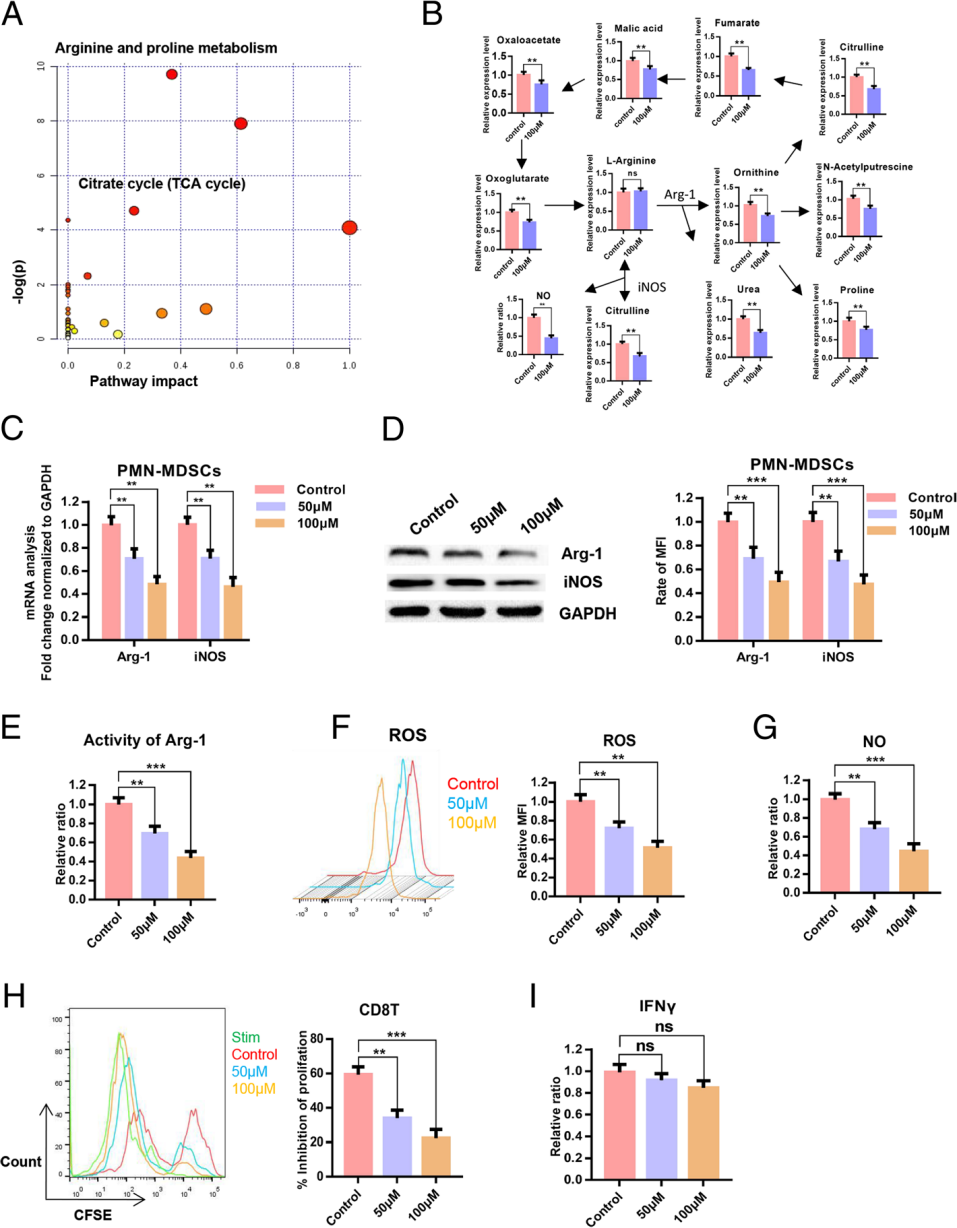
POG inhibits the immunosuppressive capacity of PMN-MDSCs without affecting the function of T-lymphocytes in vitro. **a**-**b** Results of the metabolomics analysis of B16-F10 tumour-bearing PMN-MDSCs after POG treatment. **c** qRT-PCR detection of the effect of POG on iNOS and Arg-1 expression levels of PMN-MDSCs. **d** Western blot analysis of the effect of POG on iNOS and Arg-1 expression levels in PMN-MDSCs. **e-g** Effect of POG on ARG1 activity (E), ROS (F) and NO (**g**) production of the PMN-MDSCs. **h** Effect of POG on the ability of PMN-MDSCs to inhibit the proliferation of CD8 T-lymphocytes. **i** Effect of POG on the IFN-γ content in CD8 T-lymphocytes. The pooled data from three independent experiments are shown. All data are represented as the mean ± SD. * *p* < 0.05, ** *p* < 0.01, ***p < 0.001, ****p < 0.0001

To verify the inhibitory effect of POG on PMN-MDSCs, we evaluated the effects of POG on apoptosis and proliferation of PMN-MDSCs, CD8 T-lymphocytes and B16-F10 cells. The results showed that POG exhibited no cytotoxic effect on PMN-MDSCs, CD8 T-lymphocytes and B16-F10 cells. However, POG could specifically inhibit the proliferation of PMN-MDSCs (Fig. 4a–b). To detect the key cellular signalling pathways affected by POG, we performed proteomics and metabolomics analysis. Proteomic profile changes in the POG-treated PMN-MDSCs were analysed. Consistent with the results of the upregulated proteins of B16-F10 tumour-bearing PMN-MDSCs, the results of GO analysis showed that the functions of cell proliferation, oxidation–reduction process, nucleoside metabolic process-related biological processes (Fig. 4c), NADH dehydrogenase activity, oxidoreductase activity and ATP binding-related molecule function of the PMN-MDSCs were downregulated after POG treatment (Fig. 4d). KEGG analysis results showed that after POG treatment, the RNA polymerase, biosynthesis of amino acids and metabolic pathways of the PMN-MDSCs were downregulated (Fig. 4e). GSEA analysis also revealed that POG mainly inhibits the cell cycle of PMN-MDSCs (Fig. 4f). Furthermore, we analysed the protein interaction in the downregulated genes after POG treatment with the STRING database. The results indicated that the downregulated genes after POG treatment were mainly related to cell metabolism (Fig. 4g). These findings showed that POG could inhibit the proliferation and metabolism of PMN-MDSCs. The metabolomics results showed that POG mainly inhibited arginine and proline metabolism and the citrate cycle in the PMN-MDSCs. Through pathway analysis, we found that after POG treatment, the metabolic pathways of arginine into ornithine and citrulline regulated by ARG-1 and iNOS were downregulated, and the metabolism of citrulline and ornithine further affected the TCA cycle (Fig. 5a–b).

### POG inhibits the immunosuppressive capacity of PMN-MDSCs without affecting the function of CD8 T-lymphocytes in vitro

To verify the inhibitory effect of POG on arginine metabolism in B16-F10 tumour-bearing PMN-MDSCs, we used qRT-PCR and Western blot analysis to examine the effect of POG on the expression of iNOS and Arg-1 in PMN-MDSCs. The results showed that POG decreased the expression of Arg-1 and iNOS in PMN-MDSCs (Fig. 5c–d). We then examined the ARG1 activity, ROS and NO levels of the PMN-MDSCs after POG treatment. The findings revealed that POG inhibited ARG1 activity, the production of ROS and the production of NO in PMN-MDSCs (Fig. 5e–g). To evaluate the effect of POG on the immunosuppressive capacity of PMN-MDSCs, we co-cultured the control and POG-treated PMN-MDSCs with CD8 T-lymphocytes at 1:1 for 48 h to detect the proliferation of CD8 T-lymphocytes. The results indicated that POG inhibited the inhibitory activity of the PMN-MDSCs on T-lymphocyte proliferation (Fig. 5h). To evaluate the effect of POG on CD8 T-lymphocyte function, we co-cultured CD8 T-lymphocytes with POG in T-lymphocyte medium for 48 h to examine the production of IFN-γ in T-lymphocytes. The results showed that POG did not influence the production of IFN-γ in CD8 T-lymphocytes (Fig. 5i).

### POG exerts a dose-dependent antitumour effect and improves the immunosuppressive microenvironment of tumours

**Fig. 6:**
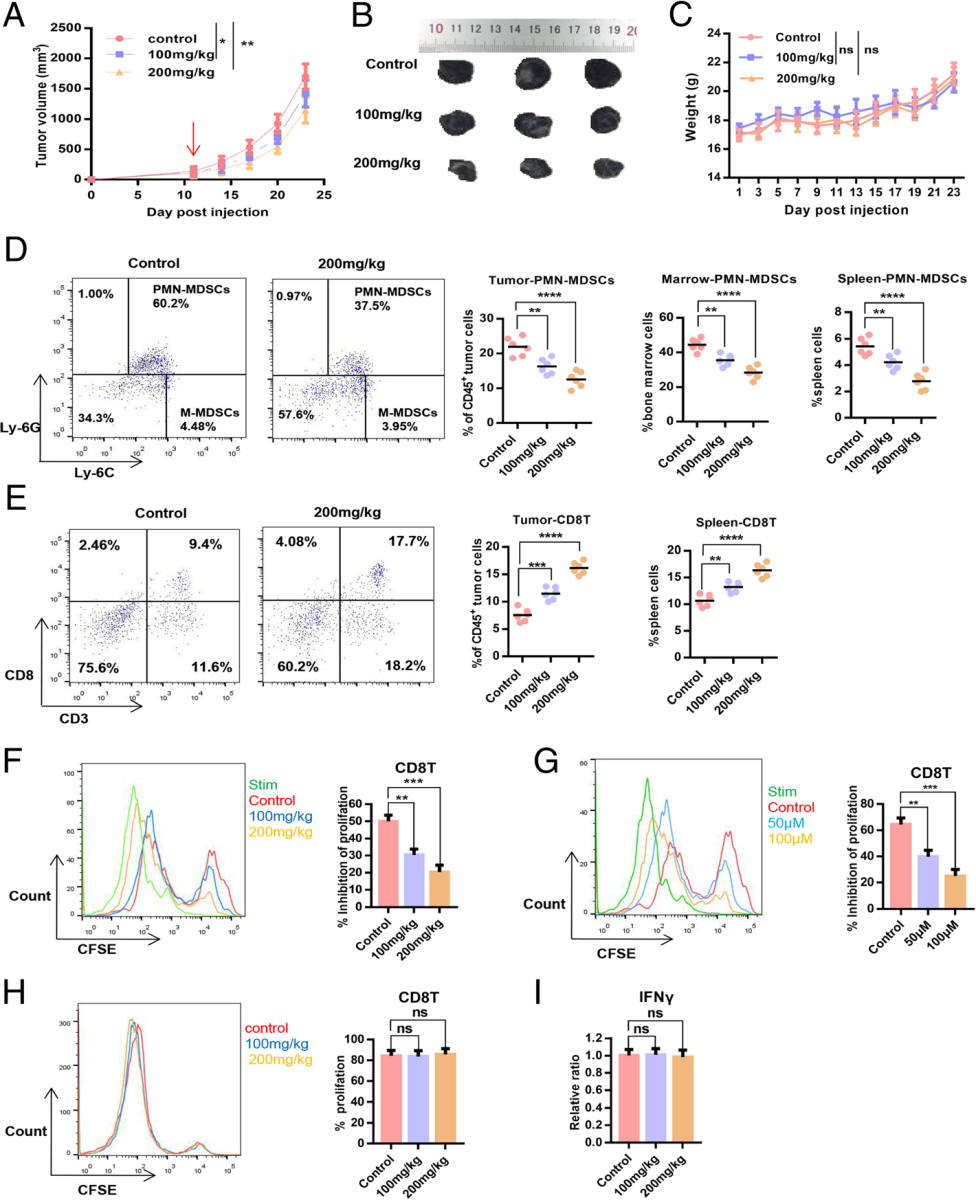
POG exerts a dose-dependent antitumour effect and improves the immunosuppressive microenvironment of tumours. **a** Tumour growth curves of B16-F10 tumour-bearing mice after POG treatment (*n* = 6). **b** Representative tumour images of control and POG-treated B16-F10 tumour-bearing mice (*n* = 6). **c** Body weight of B16-F10 tumour-bearing mice after POG treatment (*n* = 6). **d** Dotplots of live, CD45^+^CD11b^+^ cells in the tumours of control and POG-treated B16-F10 tumour-bearing mice (left panels) and proportion of PMN-MDSCs (CD11b^+^Ly6G^+^Ly6C^low^) in bone marrow, spleen and CD45^+^ cells from tumours of control and POG-treated B16-F10 tumour-bearing mice (*n* = 6) (right charts). **e** Dotplots of live, CD45^+^ cells in the tumours of control and POG-treated B16-F10 tumour-bearing mice (left panels) and proportion of CD8 T-lymphocytes (CD3^+^CD8^+^) in spleens and CD45^+^ cells from tumours of control and POG-treated B16-F10 tumour-bearing mice (*n* = 6) (right charts). **f-g** Ability of PMN-MDSCs sorted from bone marrow (**f**) or tumours (**g**) of control and POG-treated B16-F10 tumour-bearing mice to inhibit CD8 T-lymphocyte proliferation (*n* = 6). **h** Proliferation of CD8 T-lymphocytes sorted from the spleens of control and POG-treated B16-F10 tumour-bearing mice (*n* = 6). **i** IFN-γ content of CD8 T-lymphocytes sorted from the spleens of control and POG-treated B16-F10 tumour-bearing mice (*n* = 6). The pooled data from three independent experiments are shown. All data are represented as the mean ± SD. * *p* < 0.05, ** *p* < 0.01, ***p < 0.001, ****p < 0.0001

We established the B16-F10 subcutaneous tumour model in C57BL6 mice to evaluate the dose-dependent effect of POG on B16-F10 primary tumour growth and the tumour immunosuppressive microenvironment. The results showed that POG resulted in significant inhibition of tumour growth dose-dependently, and 200 mg/kg exerted no significant effect on the body weight of mice (Fig. 6a–c). To investigate the dose-dependent effect of POG on the immunosuppressive microenvironment, the proportion of PMN-MDSCs and CD8 T-lymphocytes in the spleens, bone marrow and tumours of mice in the control group and the POG-treated group was compared. The results showed that the proportion of PMN-MDSCs in bone marrow, spleen and CD45^+^ cells from tumours was reduced, and the proportions of CD8 T-lymphocytes in spleens and CD45^+^ cells from tumours were increased dose-dependently after treatment with POG (Fig. 6d–e). To investigate the effect of POG on the immunosuppressive ability of PMN-MDSCs, we co-cultured PMN-MDSCs sorted from the bone marrow and tumours of the control and POG-treated B16-F10 tumour-bearing mice with CD8 T-lymphocytes at 1:1. CD8 T-lymphocyte proliferation was examined after 48 h. The results showed that the immunosuppressive ability of PMN-MDSCs from the bone marrow and tumours in the POG-treated group considerably decreased relative to that of the control group in a dose-dependent manner (Fig. 6f–g). To assess the effect of POG on CD8 T-lymphocyte proliferation and function, we sorted the CD8 T-lymphocytes from the spleens of the control and POG-treated B16-F10 tumour-bearing mice. After 48 h, we examined the proliferation and IFN-γ production ability of spleen CD8 T-lymphocytes. The results showed that POG did not affect the proliferation and IFN-γ production ability of the spleen CD8 T-lymphocytes (Fig. 6h–i). These results showed that POG selectively inhibited the proliferation and immunosuppression of PMN-MDSCs and improved the immunosuppressive microenvironment of B16-F10 tumour-bearing mice, thereby inhibiting tumour growth in vivo in a dose-dependent manner.

### POG enhances the antitumour effect of PD-1 inhibitor in B16-F10 and 4 T1 mouse tumour models

**Fig. 7:**
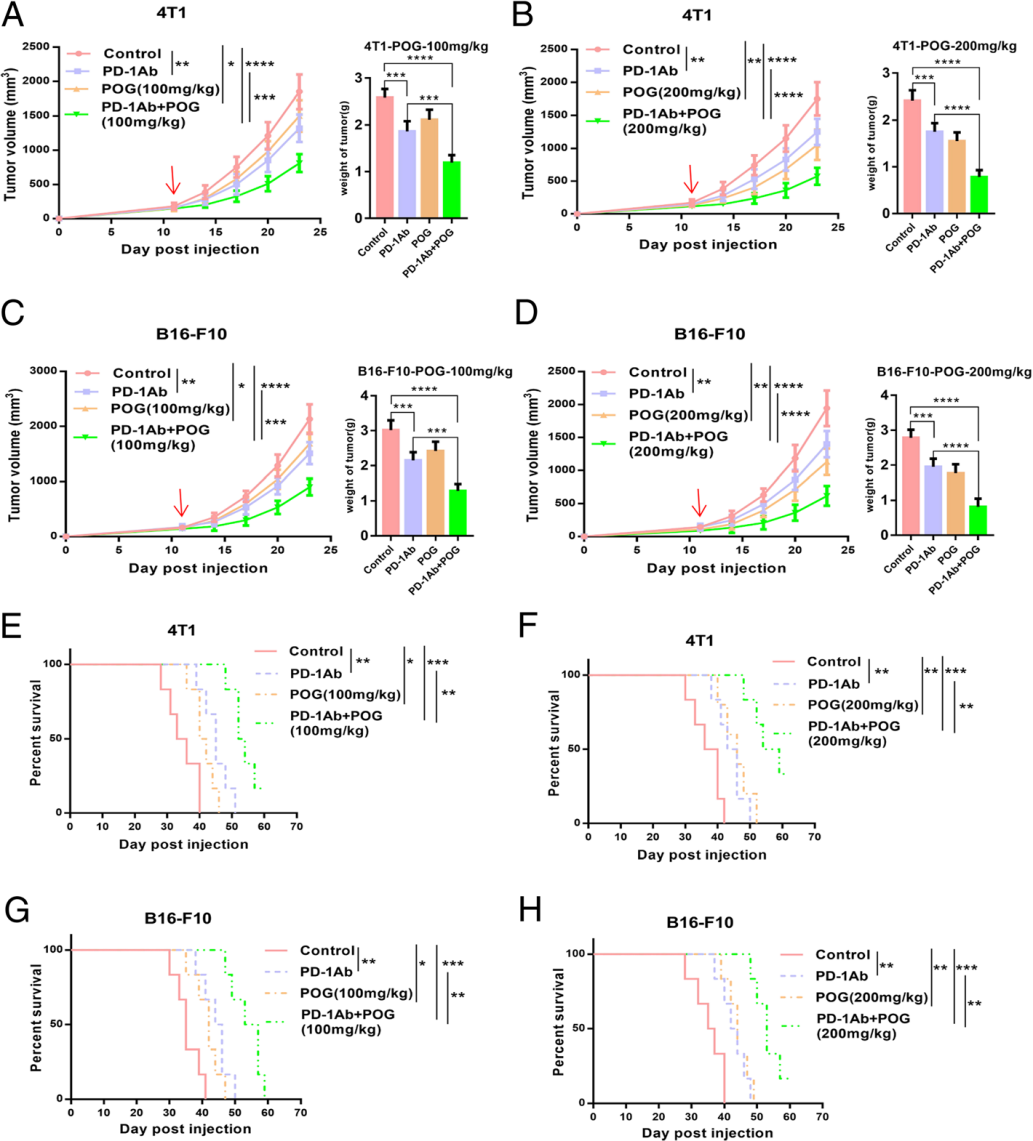
POG enhances the antitumour effect of PD-1 inhibitor in mouse tumour models of B16-F10 and 4 T1. **a**-B) The tumour growth curves of 4 T1 tumour-bearing mice after POG and anti-PD-1 antibody (alone or in combination) treatment (*n* = 6). **c**-**d** The tumour growth curves of 4 T1 tumour-bearing mice after POG and anti-PD-1 antibody (alone or in combination) treatment (*n* = 6). **e**-**f** Survival rate of 4 T1 tumour-bearing mice with POG and anti-PD-1 antibody (alone or in combination) treatment (*n* = 6) **g**-**h** Survival rate of B16-F10 tumour-bearing mice with POG and anti-PD-1 antibody (alone or in combination) treatment (*n* = 6). The pooled data from three independent experiments are shown. All data are represented as the mean ± SD. * *p* < 0.05, ** *p* < 0.01, ***p < 0.001, ****p < 0.0001

Given that POG reduced PMN-MDSCs in the bone marrow and tumours and increased CD8 T-lymphocytes in the spleens and tumours of the B16-F10 tumour-bearing mice, we hypothesised that POG enhances the antitumour effect of the PD-1 inhibitor. We established mouse B16-F10 subcutaneous and 4 T1 in situ tumour models. The results showed that the combination of POG and PD-1 mAb group showed better antitumour effects than did the POG and PD-1 mAb groups. The combination index [30] of POG (100 mg/kg) and POG (200 mg/kg) with PD-1 mAb was 1.27 and 1.32 in the B16-F10 tumour model and 1.23 and 1.21 in the 4 T1 tumour model, respectively (Fig. 7a–d). The combination group also showed the best ability to prolong survival time of B16-F10 and 4 T1 tumour-bearing mice compared with the other groups (Fig. 7e–h). These results indicated that POG and PD-1 inhibitors exhibited synergistic antitumour effects.

## Discussion

MDSCs comprise a highly immunosuppressive population of tumour-infiltrating immature myeloid cells that contribute to tumour immune escape by inhibiting cytotoxic T-lymphocyte proliferation and driving T regulatory cell induction [31, 32]. MDSCs penetrate the entire tumour and are correlated with tumour size and malignancy. Therefore, targeting MDSCs is an important therapeutic strategy for tumour immunotherapy.

In the present study, we found that PMN-MDSCs heavily accumulated in the spleens and bone marrow of B16-F10 tumour-bearing mice, and the proliferation, metabolism and immunosuppression of B16-F10 tumour-bearing PMN-MDSCs increased. We selected the top 10 key proteins, namely, Eprs, Gart, Umps, Paics, Atp5o, Hadha, Dld, Mrpl4, Rpl8 and Mrpl13, in the upregulated KEGG pathways of B16-F10 tumour-bearing PMN-MDSCs as targets to screen the natural inhibitors of PMN-MDSCs from the traditional Chinese Medicine library (20000 compounds). The top 10 key proteins are mainly RNA and ATP binding proteins involved in protein translation, amino acid metabolism and ATP synthesis. Among these proteins, Eprs is an ATP binding protein involved in the metabolism of L-glutamate and L-proline, Dld is an E3 component of the three alpha-ketoacid dehydrogenase complexes with electron transfer activity, and Atp5po participates in the synthesis of ATP [33, 34].

Finally, we found that POG could bind well to the key proteins in these pathways, inhibit B16-F10 primary tumour growth and improve the immunosuppressive microenvironment of B16-F10 tumour-bearing mice. POG is a chromone extracted from Saposhnikovia root [35]. POG has been reported to inhibit the production of TNFα, IL-1β and IL-6 in Raw 264.7 cells by inhibiting the activation of MAPK and NF-κB signalling pathways and reducing serum TNFα, IL-1β and IL-6 in vivo [36, 37]. In addition, POG could dose-dependently inhibit the expression of iNOS, COX-2 and PGE2 by suppressing the activation of JAK2/STAT3 signalling in vitro and in vivo [37, 38].

Mechanically, POG reduces the content of ornithine and citrulline in PMN-MDSCs by inhibiting the expression of Arg-1 and iNOS, which further inhibits polyamine production and the TCA cycle and ultimately inhibits the proliferation, metabolism and immunosuppressive ability of cells [39, 40]. As mentioned above, MDSCs might have partly limited immune checkpoint inhibitors, and combination therapies increase the response rates of PD-1/PD-L1 inhibitors [41–43]. In the present study, we found that POG treatment enhanced the effect of anti-PD-1 immune checkpoint blockade in mouse tumour models of B16-F10 and 4 T1. This result provides a new direction for improving the response rate of PD-1 pathway blockade.

We successfully screened POG from the traditional Chinese Medicine library (20000 compounds) as a PMN-MDSC inhibitor. The POG exhibits a good synergistic antitumour effect with PD-1 inhibitors. The synergistic antitumour effect of POG and PD-1 inhibitors offers a reasonable basis for future clinical combination therapy of POG and PD-1 inhibitors to overcome the low response rate and recurrence of PD-1 in clinical practise.

### Additional files

Additional file 1: The structures of the traditional Chinese medicines from TCM Database@Taiwan. (SDF 93000 kb)

## Data Availability

All datasets used and/or analysed during the current study are available from the corresponding author on reasonable request.

## Appendix

**Table 1:** The structure of 10 candidate inhibitors

Compounds name	Atp5o	Dld	Eprs	Gart	Hadha	Mrpl4	Umps	Mrpl13	Paics	Rpl8
	-6.781	-7.363	-5.189	-5.359	-5.97	-4.184	-5.189	-4.987	-4.185	-4.748
	-5.86	-5.327	-4.954	-4.895	-5.143	-4.993	-5.329	-6.568	-4.862	-4.333
	-5.255	-4.54	-4.73	-4.619	-5.851	-5.679	-4.995	-4.248	-4.379	-4.233
	-4.92	-4.667	-5.369	-4.218	-5.58	-5.089	-4.478	-5.008	-4.221	-4.52
	-4.588	-4.085	-4.408	-4.691	-5.101	-4.375	-4.682	-5.208	-5.517	-4.324
	-4.465	-5.814	-5.219	-4.761	-4.96	-4.089	-4.01	-5.333	-4.105	-4.152
	-5.603	-4.096	-5.514	-4.437	-4.492	-4.523	-4.39	-4.279	-4.772	-4.229
	-4.701	-5.962	-4.701	-5.174	-5.389	-5.373	-5.076	-5.673	-5.234	-4.734
	-4.042	-5.414	-5.311	-4.452	-5.003	-4.575	-4.349	-4.989	-4.155	-4.204
	-4.167	-5.221	-4.634	-4.593	-5.418	-4.593	-4.085	-4.422	-4.652	-4.565
